# Silver Eco-Solvent Ink for Reactive Printing of Polychromatic SERS and SPR Substrates

**DOI:** 10.3390/s18020521

**Published:** 2018-02-09

**Authors:** Mavlavi Dustov, Diana I. Golovina, Alexander Yu. Polyakov, Anastasia E. Goldt, Andrei A. Eliseev, Efim A. Kolesnikov, Irina V. Sukhorukova, Dmitry V. Shtansky, Wolfgang Grünert, Anastasia V. Grigorieva

**Affiliations:** 1 Department of Materials Science and Department of Chemistry, Lomonosov Moscow State University, Leninskie gory 1, bld. 73, 119991 Moscow, Russia; mr.mavlavi@mail.ru (M.D.); gol_dia@mail.ru (D.I.G.); a.yu.polyakov@gmail.com (A.Y.P.); chekanova@gmail.com (A.E.G.); eliseev@inorg.chem.msu.ru (A.A.E.); efimkolesnikov12@gmail.com (E.A.K.); 2 Skolkovo Institute of Science and Technology, Skolkovo Innovation Center, bld. 3, 143026 Moscow, Russia; 3 National University of Science and Technology MISiS, Leninsky prospect 4, 119049 Moscow, Russia; irina_btnn@mail.ru (I.V.S.); shtansky@shs.misis.ru (D.V.S.); 4 Department of Chemistry and Biochemistry, Ruhr-Universität Bochum, Universitätsstraße 150, Bochum 44801, Germany; w.gruenert@techem.rub.de

**Keywords:** reactive ink, photoreaction, silver nanostructures, surface-enhanced Raman scattering, plasmon resonance

## Abstract

A new reactive ink based on a silver citrate complex is proposed for a photochemical route to surface-enhanced Raman spectroscopy active substrates with controllable extinction spectra. The drop-cast test of the ink reveals homogeneous nucleation of silver and colloid particle growth originating directly from photochemical in situ reduction in droplets, while the following evaporation of the deposited ink produces small nano- and micron-size particles. The prepared nanostructures and substrates were accurately characterized by electron microscopy methods and optical extinction spectroscopy. Varying the duration of UV irradiation allows tuning the morphology of individual silver nanoparticles forming hierarchical ring structures with numerous “hot spots” for most efficient Raman enhancement. Raman measurements of probe molecules of rhodamine 6G and methylene blue reached the largest signal enhancement of 10^6^ by the resonance effects.

## 1. Introduction

Within the last decades, surface-enhanced Raman scattering (SERS) has been developed from a fantastical phenomenon [1] to a powerful analytical tool, capable of detecting single-molecules and identification of a great variety of analytes in environmental and biomedical samples [2]. Silver and gold nanoparticles of various shapes and sizes or nanoscale roughened substrates demonstrating strong plasmon resonances in the visible range are widely employed as mediators for the Raman signal enhancement [3]. However, the widespread incorporation of SERS in viable sensing platforms still requires cost-effective techniques for a uniform and reproducible mass-production of SERS-active chips. Polychromatic SERS substrates, i.e., substrates with multiple plasmon resonances in the visible range, are the most advanced and demanded products appropriate for different kinds of analytes. This is because they allow for the surface enhanced resonant Raman scattering (SERRS) effect, when the wavelength of a substrate plasmon resonance coincides with the wavelength of laser excitation and the maximum of the analyte optical absorption spectrum.

Different printing techniques were recently reported to be effective for the production of SERS substrates. For instance, Jiang et al. [4] printed gold nanorods onto flexible paper substrates to vary plasmonic characteristics of the films mechanically. Inkjet printing is also an attractive technology to fabricate large-scale patterns due to its advantages of low cost, of the efficient use of materials and of waste elimination [5,6,7,8]. As reported recently [9,10,11], viscosity *υ* of inks for inkjet printers varies in a wide range of 2.5–5.6 mPa·s. The surface tension *σ* should be larger than 25 mN/m. Smart combination of *σ* and *υ* dimensions allows for uniform deposition using specific types of the nozzle. Recently, inkjet printing of pre-synthesized silver and gold colloids was employed for the design of highly reproducible SERS-active substrates [12,13,14,15]. In a recent manuscript by Betz et al. [16], the authors declared inkjet printing as one of most powerful techniques for SERS substrate fabrication. Wu et al. [17] suggested that the inks explored for inkjet printing may be appropriate also for other printing techniques.

It should indeed be considered if the routine printing techniques, namely, screen-printing and pin-printing, which are widely used in microchip processing, are also promising for polychromatic SERS pattern formation. Screen printing is technologically the most simple and low-cost process. However, its potential for the deposition of platforms of plasmon spots, for example, for SERS analysis, has not yet been demonstrated. Depending on the instrument used for the screen printing process, *υ* and *σ* parameters of the depositing inks could vary in wide ranges [18]. Namely, for screen-printing *σ* should be less than 40 mN/m if deposition performs onto polymer textile substrates [19] but could be larger if deposition performs onto glass slides substrates. The kinematic viscosity in screen-printing process should be 1.5–6 mPa·s. For pin-printing *σ* could be 24–63 mN/m and *υ* as high as 2–7 mPa·s. The more rigid and less wettable tip in pin-printing requires more viscous inks up to 12 mPa·s.

However, when these printing techniques are to be used for the production of SERS active patterns, one needs to concentrate the plasmonic nanoparticles by multi-run printing to build up structures with a proper number of “hot spots”. Another approach is preparation [20] and use of highly concentrated inks which, however, easily clog the ink feed systems and nozzles. Therefore, the development tends towards reactive printing methods [21], which are expected as the next step in the evolution of SERS chip printing. Most existing techniques require inks based on true solutions of silver precursors (such as silver nitrate, AgNO_3_) which do not contain any pre-synthesized colloids. Therefore, the ink concentration can be in principle substantially increased. The other reactants, e.g., reducing agents, can be admixed to the main ink in the printing head (which requires rather complicated hardware), printed in a next run or pre-deposited on the substrate by another technique such as spin-coating or impregnation [21,22]. Post-printing thermal treatment leading to chemical deposition of inks was proposed by Farraj et al. for production of conductive copper patterns on flexible plastics [23]. A comprehensive recent review by Chiolerio et al. covered the numerous methods for making polychromatic SERS substrates [15].

To the best of our knowledge, there are only very few articles reporting inks for reactive inkjet printing of SERS-active chips so far. In Refs. [24,25,26], authors applied silver nitrate inks for printing of SERS-active silver layers on porous silicon surfaces. By optimizing the nanostructure morphology in terms of densely packed silver particles, huge Raman enhancements (>10^8^) were obtained [25]. However, silver nitrate inks, although in principle without risks for printing devices and subsystems, need very specific storage conditions to prevent premature formation of silver particles. In addition, reduction of silver nitrate actually leads to larger silver particles instead of nano-scale crystallites. Therefore, ammonia-based silver complexes are preferred for nano-scale particles [13]. However, even these require the presence of stabilizers (polymer surfactants) to prevent the formation of silver mirrors, which would interact with aldehydes or alcohols and thus interfere with applications involving complex solvents.

Here, we report a novel composition of silver ink for the preparation of polychromatic silver SERS-active chips using a Ag^I^ citrate complex as a precursor for polycolor nanocoating. This silver complex has been earlier applied as an ink compound proposed for microelectronics for high-conductivity features printing being an appropriate alternative to the less stable acetate complex and to a group of Tollens’ reagents [27]. Without stabilizing polymer surfactant, the widely used ammonia-based silver complexes form metal mirrors interacting with aldehydes or alcohols, which interferes with their further application in complex solvents. As the deposited component, we propose silver citrate complex as an ink component, which is appropriate for SERS substrate preparation and undergoes an easy-to-handle reduction induced by UV irradiation after the drop-cast or printing at the substrate. The hypothetic scheme for polychromic SERS platform production is given in Figure S1.

## 2. Materials and Methods

AgNO_3_ salt was purchased from Sigma-Aldrich (ACS reagent grade, ≥99.0%). All other reactants were of analytical grade. The aqueous solutions were prepared using ultra-purified water (Milli-Q). All glassware and stirring bars used for preparation of silver precursors and nanoparticles was rinsed with 98% HNO_3_ to remove any possible silver seeds and reductants.

To prepare the mentioned Ag^I^ complex for the reactive ink, we used the water-based technique described elsewhere [28]. The silver citrate complex was obtained in a double excess of citric acid to silver salt. First, 0.17 g of AgNO_3_ was dissolved in 30 mL of purified water. Then, 0.5 mL of 25% (NH_3_)_aq_ was added:

AgNO_3_ + 3(NH_3_)_aq_ + H_2_O → [Ag(NH_3_)_2_]OH + NH_4_NO_3_

The as-prepared solution was mixed with 20 mL of 1 M citric acid (H_3_C_6_H_5_O_7_) observing silver(I) citrate spontaneous precipitation and following dissolution:

3[Ag(NH_3_)_2_]OH + 3H_3_Cit → Ag_3_Cit↓ + 2(NH_4_)_3_Cit + 3H_2_O

Ag_3_Cit + nH_3_Cit → [Ag_3_(Cit)_n+1_]^3n−^ + 3n H^+^

Cit = citrate anion (C_6_H_5_O_7_^3−^), n = 1–6.

This synthesis resulted in 50 mL of aqueous containing 10 mM Ag^+^ ions.

From this photosensitive compound, different inks based on a double-component (water/ethylene glycol (EG)) solvent were made by mixing more concentrated aqueous solutions with ethylene glycol and distilled water. The citrate obtained was dissolved in the water/EG mixtures previously cooled to 4 °C, and stored in the dark. The H_2_O:EG ratio was varied (see Supplementary Materials) to find which physicochemical characteristics would be most efficient for the printing process.

To analyze the photochemical reduction, 1 mL aliquot of the prepared ink was irradiated by UV at 312 nm for different durations between 2 and 30 min. The experiment was performed in quartz cuvettes using a Vilber Lourmat VL-6 MC lamp.

UV-vis absorption spectra were registered using a UV-vis spectrometer Lambda 950 (Perkin-Elmer) with an attached diffuse reflectance accessory. Measurements have been performed in the spectral range of 250–1000 nm with 1 nm step and a scanning rate of 2 nm/s.

Silver colloids obtained by photochemical silver citrate decomposition were characterized by transmission electron microscopy (TEM) combined with electron diffraction (ED) using a LEO 912 AB OMEGA microscope (Carl Zeiss, Jena, Germany) at an accelerating voltage of 100 kV. For statistical analysis of the particles, diameters or linear extensions of about three hundred nanoparticles were measured. For TEM analysis of silver nanoparticles formed on quartz slides, the same equipment was used. Dry nanoparticle films were scraped off the slides and transferred to the copper grids.

Silver substrates prepared by photoreduction of the silver citrate ink on the quartz slide were also analyzed with LEO Supra 50 VP scanning electron microscope (Carl Zeiss) coupled with the energy dispersive X-ray microanalysis (Oxford Instruments Ltd., Abingdon, UK). An optimal accelerating voltage of 3 kV was used to reduce sample charging. The working distance was 4 mm, and an InLens detector was used. Images were taken at magnifications of 400–80000×.

XPS was measured with a Leybold LHS 10 spectrometer equipped with a EA 10/100 multichannel analyzer, using Mg Ka irradiation (1253.6 eV, 12 kV, 20 mA) for excitation.

SERS experiments were performed with an InVia Raman confocal microscope (Renishaw Inc., Wotton-under-Edge, UK). Two different lasers were applied for excitation—a 50 mW 514.4 nm argon laser combined with a power neutral density filter (1–10%) and a 20 mW 632.8 nm Ne-He laser. All spectra were collected with a confocal Leica DMLM microscope (resolution up to 2.5 μm) using 20× objective lens with 10 s acquisition time and 100 accumulations. The diffraction grating was 2400 lines/mm, and the resolution of the CCD camera was 1024 × 256 pixels. A silicon (100) single crystalline wafer was used for calibration.

Raman mapping experiments were performed on Renishaw InVia spectrometer equipped with Leica DMLM optics (50× objective) using 50 mW 514.4 nm Ar laser. The scans we collected in Streamline accumulation mode with line-focused laser beam with a length of ~50 mkm and a thickness below 1 mkm. The spectra were registered using 2400 L/mm grating and Peltier-cooled 1024 × 568 detector, resulting in point-to-point resolution of 1.2 mkm and spectral resolution of ~1 cm^−1^. Total accumulation time for each point equaled 100s. Analysis of spectral data was carried out using Wire 3.4 Renishaw software. Pseudo-Voigt fitting of the peak positioned at ~1365 cm^−1^ was performed in the range of 1280–1420 cm^−1^ using third order baseline subtraction and final tolerance factor for each spectrum of 0.01.

## 3. Results and Discussion

Important quality criteria of the synthesized inks, such as thermal stability, kinematic viscosity υ and surface tension *σ*, were studied prior to further photodecompositon tests (Table S1). All the surface tension characteristics measured for silver compositions were in the reasonable range (from 53.5 mN/m to 65.5 mN/m) for its screen-printing onto cleaned glass surface.

**Scheme 1:**
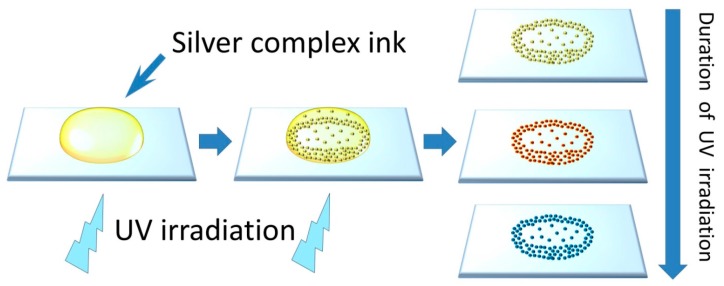
Principal scheme of production of SERS-active substrates by photochemical reaction in silver complex ink.

**Figure 1:**
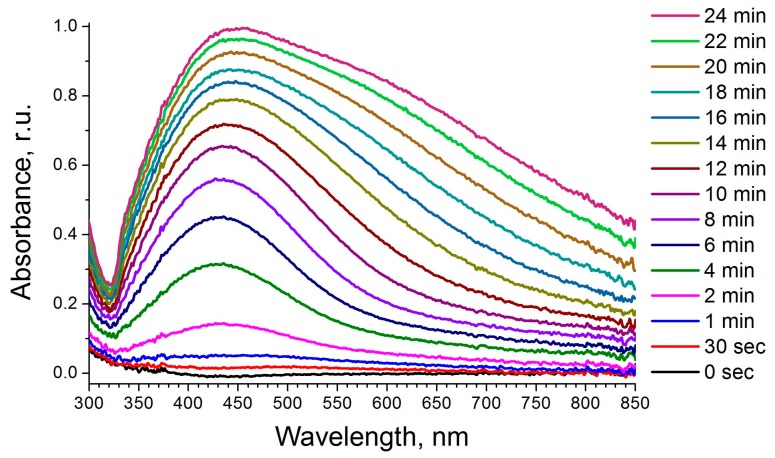
UV-vis spectra of silver(I) citrate complex in 1:1 H_2_O:EG solvent after 312 nm illumination for 2–24 min.

The conversion of silver ink to polychromatic silver nanoparticles was performed through UV-induced reduction (Scheme 1). First, the photodecomposition of a model ink was studied in solution rather than on a substrate. All the silver citrate ink solutions became tinted after illumination by 312 nm UV-light for 2 min as well as because of the surface plasmon resonance effect (Figure 1). Short illumination (2–10 min) produced mostly yellow colored colloids while 16 min UV-illumination resulted in red colloids. Longer illumination durations (up to 24 min) led to purple sol color changing to dark blue. The UV-Vis-NIR absorption spectra revealed just one strong maximum at about 440 nm after 6–12 min illumination. At longer illumination times, an additional shoulder appeared and became red-shifted forming a separate maximum at 630 nm for ink drops illuminated for 24 min (Figure 1). The observed spectral effect could be attributed both to nanoparticles growth [29] and also to formation of platelet-like nanoparticles which could be observed in presence of ethylene glycol and citrate molecules [13]. Thus, by varying the time of UV-illumination of silver citrate complex, it is possible to change the spectral range of plasmon resonance easily. An easy tuning of illumination time produces the maximal efficiency of signal enhancement due to the SERRS effect.

It was also important to analyze the morphology of silver nanoparticles resulting from the UV-induced reduction if silver citrate. Transmission electron microscopy was used to study particle size distribution in the colloids and the growth of the colloidal particles resulting from the aging effect [30]. For the analysis, sol droplets were placed on carbon-covered copper grids, rapidly pat dried and then kept in a vacuum chamber for the complete evaporation of the solvent.

**Figure 2:**
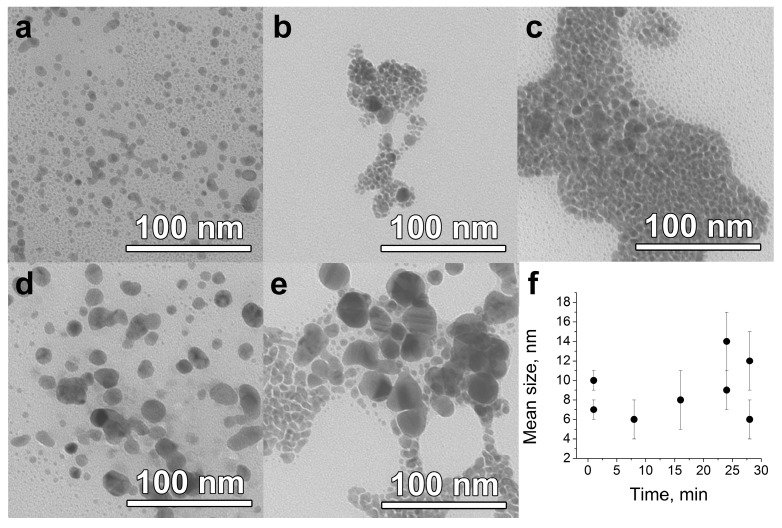
Transmission electron microscopy (TEM) micrographs of silver colloids synthesized via UV-induced photochemical reduction of silver citrate in water polyol 1H_2_O:1EG media. Time of UV illumination: (**a**) 1 min; (**b**) 8 min; (**c**) 16 min; (**d**) 24 min; and (**e**) 28 min. Mean particle size (estimated from >300 particles as visualized by TEM) vs. duration of UV illumination is plotted (**f**). Note that 24–28 min of UV irradiation results in bimodal particle size distribution.

Figure 2 shows micrographs and particle size distributions for the colloids after they had been dried on the microscope grid in vacuum. Particles with a mean diameter of 6–8 nm are typical for all the samples and, likely, correspond to the 440 nm plasmon maximum in their optical absorption spectra. The broadening of this peak and the formation of a second maximum were observed for UV-illumination times of 12 min and longer. The further prolongation of the UV-illumination resulted in broad bimodal particle size distributions with the mean size up to 14 nm and the largest particle size up to 30 nm. The largest particles and particle aggregation are suggested to cause the shoulder of the plasmonic peak and its red shift as observed in the optical spectra of the colloids (Figure 1).

**Figure 3:**
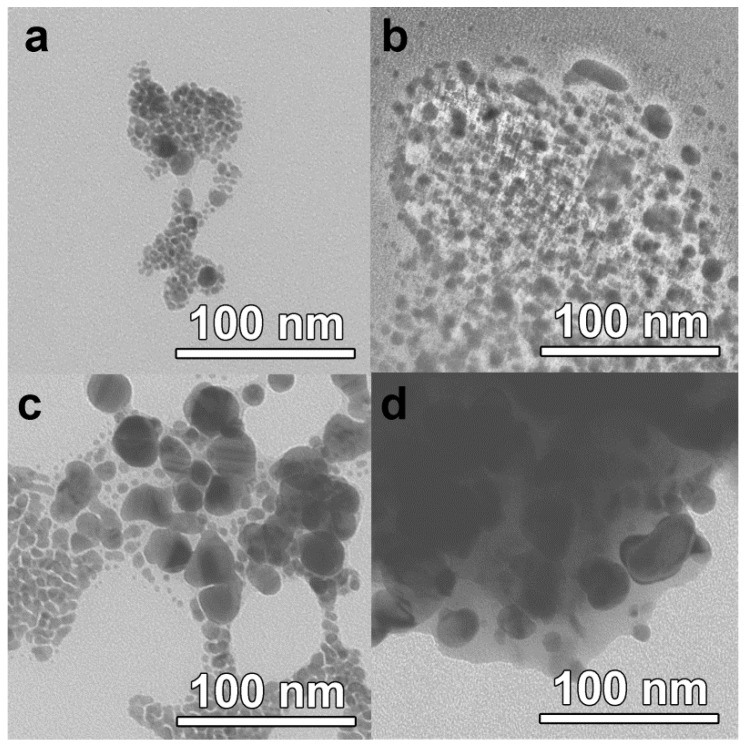
Transmission electron microscopy (TEM) images of silver nanoparticles prepared via UV-activated reduction of silver citrate complex in 1H_2_O:1EG solvent: (**a**) 8 min photoreduction reduction in bulk solution; (**b**) 8 min photoreduction on quartz slide; (**c**) 28 min photoreduction in bulk solution; and (**d**) 28 min photoreduction on quartz slide.

Similar results were obtained for samples prepared by pin-printing deposition of the ink onto planar quartz wafers. After the photochemical reaction, the droplets were dried in vacuum and then analyzed by TEM. It was demonstrated that evolution of the colloidal silver correlated well with the samples in quartz measurement cells described above (Figure 3). In Figure 3b,d, residue of EG is present.

Reduction of Ag^I^ to Ag^0^ was not easy to follow in XPS as the binding energies had an anomalous trend. Ag in the films was exactly like Ag metal, while the presence of Ag_2_O or any other Ag^I^ forms could not be excluded. Moreover, there can be no doubt that reduction occurred in the experiments because of color changes observed under illumination.

**Figure 4:**
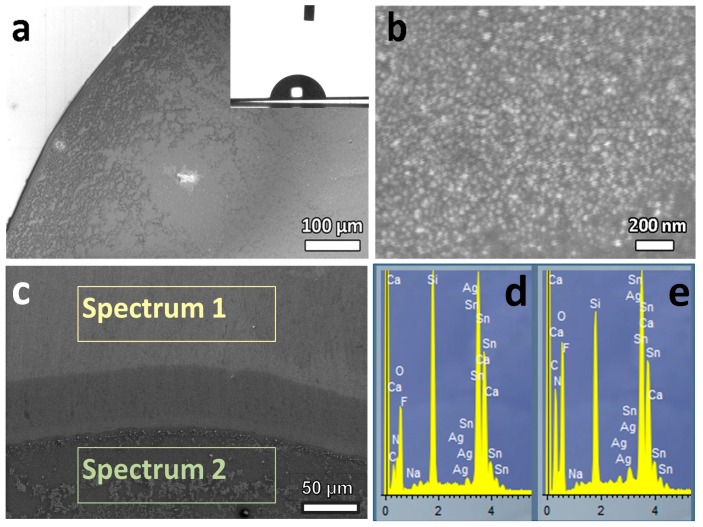
(**a**,**b**) Scanning electron microscopy (SEM) images of the hierarchical silver structures formed from silver citrate complex ink (H_2_O:EG) after 8 min UV induced reduction on the quartz substrate. (**a**) Inset: Side view of the ink droplet before drying. (**c**–**e**) Energy dispersive X-ray spectroscopy (EDX) data for the similar droplet deposited onto FTO glass substrate.

The corresponding SEM micrographs and EDX data for a vacuum dried sample are presented in Figure 4a–e, respectively. Micrographs show the typical microstructures of the spots obtained by droplet deposition with the following evaporation of solvent. This correlates with specific morphology of the dried ink spot with a shape of a Liesegang ring with rather even middle. Figure 4 demonstrates rather uniform elemental (silver) and particle size distribution within the dried spot of the ink. The elemental percentage of the silver in the sample was about 1.4 wt. % at the edge of the spot (spectrum 2) and no silver in the free zone (spectrum 1) was detected according to analysis of characteristic Ag Lα lines. In addition, both EDX scan zones show presence of oxygen, silicon, calcium, sodium and tin from FTO coverage.

The functional efficiency of the photochemically prepared silver nanostructures as Raman signal enhancers was examined on polychromatic spots of the colloidal silver which were produced via drop-casting of the colorless ink onto UV-transparent quartz wafers, further illumination by UV for different times and subsequent drying in a vacuum chamber. As photostable [31] analytes for standardized Raman enhancement tests, rhodamine 6G (R6G) and methylene blue (MB) were applied, which are among the most illumination-resistive organic dyes with absorbance covering most of the bands in visible optical range.

In series of tests using the 514.4 nm laser and 5 μL R6G droplets deposited onto drop-casted substrates (Figure 4a,b), the highest Raman intensities were achieved with larger silver particles. According to the analysis of UV-illuminated suspensions (Figure 1), the larger silver particles had a pronounced plasmon resonance peak in the 600–700 nm wavelength range providing a green-to-dark-blue color of the sols. The samples subjected to shorter UV-illumination had a plasmonic peak near 420–450 nm. Consistently, the illumination durations required for drop-casted samples correlated to those for the liquid colloid samples in quartz cells.

**Figure 5:**
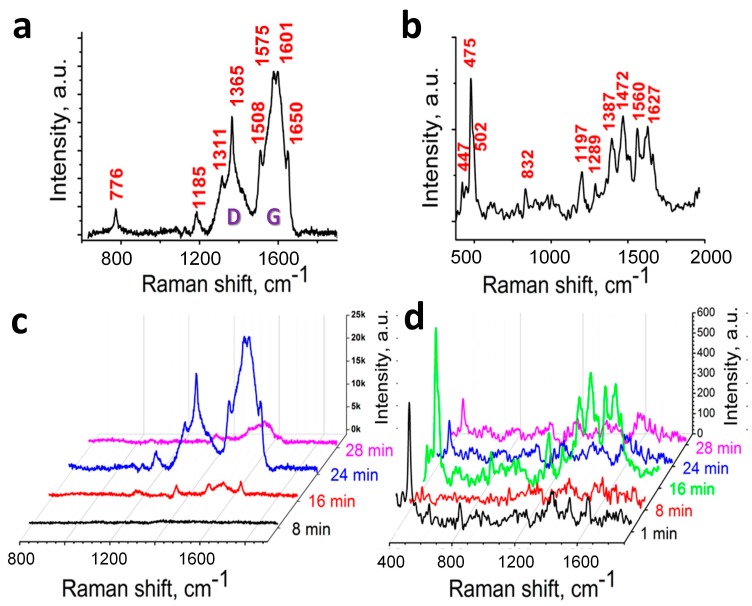
(**a**,**b**) typical SERS spectra of rhodamine 6G and methylene blue with labeled spectral lines, respectively. Carbon modes D and G at 1360 and 1590 cm^−1^, respectively, are indicated. (**c**) SERS spectra of rhodamine 6G 10^−7^ M (5 μL aliquot) at the substrates produced on quartz slides via UV-induced photochemical reduction of colorless silver ink for different times of UV illumination (514.4 nm Ar laser, 10% of 20 mW; 100 accumulations). (**d**) SERS spectra of methylene blue 10^−7^ M (5 μL aliquot) at the substrates produced on quartz slides via UV-induced photochemical reduction of silver citrate complex for different times of UV illumination (632.8 nm He-Ne laser, 0.1% of 17 mW; 100 accumulations).

The corresponding enhanced Raman signal spectra of 10^−7^ M R6G are given in Figure 5a,c. In fact, the spectra discussed here are better related to SERRS effect because the absorption spectra of both dyes partly overlap with the green and red wavelengths of exciting lasers [31]. The enhanced spectrum of R6G includes C-C stretching vibrations of the aromatic skeleton at 1650 (m), 1601 (s), 1575 (s), 1508 (s), and 1365 (s) cm^−1^ (Figure 5a). The mode at 1311 (m) cm^−1^ was related to C-O-C stretching vibrations of the carbon skeleton, and those at 1185 (w) cm^−1^ and 776 (w) to –CH_3_ methyl bending vibrations [32].

The calculation of the enhancement factor was performed for 10^−7^ M solutions because the scattering signal from a 10^−8^ M analyte aliquot was rather complex, which interfered with the exact identification of the substance. The enhancement factor calculated as

(1) $$G=I_{SERS}\cdot c_{RS}/I_{RS}\cdot c_{SERS}$$

where $c_{SERS}$ and $c_{RS}$ are concentrations of R6G in aliquots; and $I_{SERS}$ and $I_{RS}$ are Raman signal intensities of the 1365 cm^−1^ C-C vibration in corresponding spectra of R6G, which reached G ~ 10^6^.

Besides the intense analyte signal, a significant background with two maxima was observed, especially for the substrates with larger Raman enhancement. Most likely, the background is related to C-C bands of carbon D and G modes produced from the decomposition of residual silver citrate still present in the substrate. The strongest enhancement effect emphasized the presence of this admixture compound during Raman measurement.

Analysis of R6G and MB with 632.8 nm red exciting laser showed more efficient scattering for the MB dye which strongly absorbs light at this wavelength. Unlike pre-resonant MB, the red-colored R6G showed no evidence of characteristic modes in the spectra. The outstanding feature of the SERRS spectra collected with 632.8 nm He-Ne laser compared to those excited by the 514.4 nm Ar laser is the absence of obstructive wide bands of amorphous carbon (Figure 5b,d).

The most intense Raman modes of MB were found for the purple colored substrates obtained by 16 min UV illumination. With increasing silver particle size, the MB peaks became broader and indistinguishable. In addition to the identified peaks of MB molecules, several citrate Raman bands were present [32]. Strong spectral bands at 1627 s, 1560 s, 1472 s, 1191 s, and 475 s were related to characteristic skeleton stretching vibrations of MB (Figure 5b) [33]. Less intensive modes at 1387 s, 1289 m, 832 w, and 447 w should be related to the citrate SERS signal as they also appear in the spectrum of citrate absorbed on silver as reported by Sliman et al. [34]. The estimated enhancement factor for MB detection was smaller than for R6G, reaching just 10^4^.

The presence of the citrate signal in SERS spectra (Figure 5c) is also attributed to the effect of a less effective degradation of citrate anion under 632.8 nm irradiation discussed above. This correlates with absence of D and G modes characteristic for carbon and confirms the origin of amorphous carbon as green laser induced citrate decomposition. It may also be noted that UV-illumination for 24 and 28 min of the ink droplets deposited onto quartz substrates led to less active dark navy colored or bluish green colloids, which did not produce Raman signal enhancement if exposed to the He or Ne laser beam.

**Figure 6:**
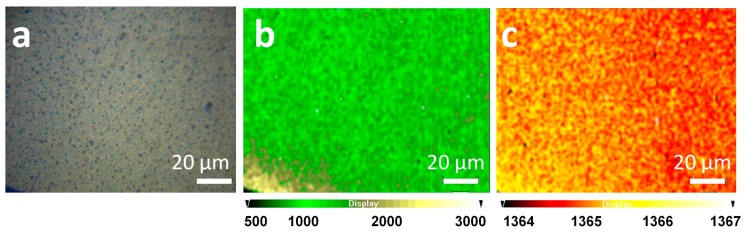
SERS images of rhodamine 6G at the substrate after 8 min of UV-illumination: optical microscopy image of substrate (**a**); and Raman maps of ~1365 cm^−1^ band: intensity (**b**); and position (**c**).

A uniform distribution of colloidal silver in the film and reproducible SERS signal was proven with SERS mapping experiment. The signal collection was carried out for R6G deposited onto the quartz substrate with silver inks after 8 min of UV illumination and vacuum drying. Figure 6a shows the optical micrograph of the film area served for the investigation. The black line in the bottom left corner is a scratch on the film used for accurate positioning of the optical signal. Full profile analysis was applied to extract position and the intensity of characteristic R6G 1365 cm^−1^ band. Figure 6b,c shows distribution in signal intensity and position for the analyzed band in the mapped area. Notably, acquired band position of 1365.1 cm^−1^ differ from profile analysis results presented elsewhere. Statistical analysis of the derived data indicates absolute band intensity dispersion of ~30% and band position of 1365.1 ± 0.7 cm^−^^1^ for 5–95% quantile. Very narrow peak position and uniformity of the signal intensity illustrates prospects of the proposed ink methodology for commercial applications.

## 4. Conclusions

In summary, the controlled UV-induced photochemical reduction of drop-casted ink based on a silver(I) citrate complex in water–polyol mixtures is proposed as an effective method for production of Raman signal enhancing substrates. The developed silver complex ink can also be proposed for reactive inkjet and other printing technologies since it possesses appropriate physicochemical parameters such as viscosity and surface tension. Through an accurate time-control of UV illumination of the deposited droplets, it is possible to initiate the nucleation and the following growth of the silver nanoparticles with the size up to 30 nm. Thus, the plasmon resonance spectra of the particles can be easily tuned to overlap with either exciting laser wavelength or absorption bands of the analyte. The most appropriate excitation energy for SERS and SERRS measurements is the lower energy of red lasers because the higher energies lead to incontrollable decomposition of citrate admixture right at the substrate. Probably, an excess of citrate anion could be reduced partly by an accurate pre-experimental washing of the substrates for better SERS signal magnitude. Reactive inks can also be utilized for fast production of SPR sensing platforms demanded for biomedical testing in modern analytical laboratories.

## Supplementary Materials

The following are available online at http://www.mdpi.com/1424-8220/18/2/521/s1, Figure S1: The hypothetic scheme for polycolor SERS platform production using photo-reactive silver complex inks; Table S1: Characteristics of silver citrate complex in water–polyol (H_2_O–EG) media of different volumetric ratios. Some details on Ink quality characterization are also provided.

## References

[1] Fleischmann M., Hendra P.J., McQuillan A.J. (1974). Raman spectra of pyridine adsorbed at a silver electrode. Chem. Phys. Lett..

[2] Brazhe N.A., Evlyukhin A.B., Goodilin E.A., Semenova A.A., Novikov S.M., Bozhevolnyi S.I., Chichkov B.N., Sarycheva A.S., Baizhumanov A.A., Nikelshparg E.I. (2015). Probing cytochrome *c* in living mitochondria with surface-enhanced Raman spectroscopy. Sci. Rep..

[3] Schlücker S. (2014). Surface-enhanced raman spectroscopy: Concepts and chemical applications. Angew. Chem. Int. Ed..

[4] Dai Z., Xiao X., Wu W., Liao L., Mei F., Yu X., Guo S., Ying J., Ren F., Jiang C. (2014). Side-to-side alignment of gold nanorods with polarization-free characteristic for highly reproducible surface enhanced Raman scattering. Appl. Phys. Lett..

[5] Fisslthaler E., Blümel A., Landfester K., Scherfd U., List E.J.W. (2008). Printing functional nanostructures: A novel route towards nanostructuring of organic electronic devices via soft embossing, inkjet printing and colloidal self assembly of semiconducting polymer nanospheres. Soft. Matter.

[6] Gates B.D. (2009). Flexible electronics. Science.

[7] Chen S.-P., Chiu H.-L., Wang P.-H., Liao Y.-C. (2015). Inkjet printed conductive tracks for printed electronics. ECS J. Solid State Sci. Technol..

[8] Petukhov D.I., Kirikova M.N., Bessonov A.A., Bailey M.J.A. (2014). Nickel and copper conductive patterns fabricated by reactive inkjet printing combined with electroless plating. Mater. Lett..

[9] Kim D., Jeong S., Park B.K., Moon J. (2006). Direct writing of silver conductive patterns: Improvement of film morphology and conductance by controlling solvent compositions. Appl. Phys. Lett..

[10] Guan Y., Tawiah B., Zhang L., Du C., Fu S. (2014). Preparation of UV-cured pigment/latex dispersion for textile inkjet printing. Colloids Surf. A.

[11] Xu L., Zhang W.W., Nagel S.R. (2005). Drop splashing on a dry smooth surface. Phys. Rev. Lett..

[12] Yang Q., Deng M., Li H., Li M., Zhang C., Shen W., Li Y., Guo D., Song Y. (2015). Highly reproducible SERS arrays directly written by inkjet printing. Nanoscale.

[13] Yorov K.E., Sidorov A.V., Polyakov A.Y., Sukhorukova I.V., Shtansky D.V., Grünert W., Grigorieva A.V. (2016). Correction: Inkjet printing of silver rainbow colloids for SERS chips with polychromatic sensitivity. RSC Adv..

[14] Restaino S.M., White I.M. Inkjet-fabricated surface enhanced Raman spectroscopy (SERS) sensors on paper for biosensing. Proceedings of the SPIE 9314, Optics and Biophotonics in Low-Resource Settings.

[15] Rajan K., Roppol I., Chiappone A., Bocchini S., Perrone D., Chiolerio A. (2016). Silver nanoparticle ink technology: state of the art. Nanotechnol. Sci. Appl..

[16] Betz J.F., Yu W.W., Cheng Y., White I.M., Rubloff G.W. (2014). Simple SERS substrates: powerful, portable, and full of potential. Phys. Chem. Chem. Phys..

[17] Wu J.F., Dai W.W., Liu J., Yang S., Zhou L., Xiao X., Jiang C., Roy V.A.L. (2015). Low-cost, disposable, flexible and highly reproducible screen printed sers substrates for the detection of various chemicals. Sci. Rep..

[18] Tawiah B., Howard E.K., Asinyo B. (2016). The chemistry of inkjet inks for digital textile printing—Review. BEST.

[19] Gilleo K. (1989). Rheology and surface chemistry for screen printing. Screenprinting.

[20] Yu W.W., White I.M. (2013). Chromatographic separation and detection of target analytes from complex samples using inkjet printed SERS substrates. Analyst.

[21] Smith P.J., Morrin A.J. (2012). Reactive inkjet printing. Mater. Chem..

[22] Kheawhom S., Foithong K. (2013). Comparison of reactive inkjet printing and reactive sintering to fabricate metal conductive patterns. Jpn. J. Appl. Phys..

[23] Farraj Y., Grouchko M., Magdassi S. (2015). Self-reduction of a copper complex MOD ink for inkjet printing conductive patterns on plastics. Chem. Commun..

[24] Chiolerio A., Virga A., Pandolfi P., Martino P., Rivolo P., Geobaldo F., Giorgis F. (2012). Direct patterning of silver particles on porous silicon by inkjet printing of a silver salt via in-situ reduction. Nanoscale Res. Lett..

[25] Virga A., Rivolo P., Descrovi E., Chiolerio A., Digregorio G., Frascella F., Soster M., Bussolino F., Marchiò S., Geobaldo F. (2012). SERS active Ag nanoparticles in mesoporous silicon: Detection of organic molecules and peptide–antibody assays. J. Raman Spectrosc..

[26] Novara C., Petracca F., Virga A., Rivolo P., Ferrero S., Chiolerio A., Geobaldo F., Porro S., Giorgis F. (2014). SERS active silver nanoparticles synthesized by inkjet printing on mesoporous silicon. Nanoscale Res. Lett..

[27] Walker S.B., Lewis J.A. (2012). Reactive silver inks for patterning high-conductivity features at mild temperatures. J. Am. Chem. Soc..

[28] Djokic S. (2008). Synthesis and antimicrobial activity of silver citrate complexes. Bioinorg. Chem. Appl..

[29] Soliveri G., Ardizzone S., Yüksel S., Cialla-May D., Popp J., Schubert U.S., Hoeppener S. (2016). Microwave-assisted silver nanoparticle film formation for SERS applications. J. Phys. Chem. C.

[30] Semenova A.A., Braze N.A., Maksimov G.V., Semenova I.A., Semenov A.P., Goodilin E.A. (2016). Plasmonic properties of aged silver hydrosols. Mendeleev. Commun..

[31] Nicolai S.H.A., Rubim J.C. (2003). Surface-enhanced resonance raman (SERR) spectra of methylene blue adsorbed on a silver electrode. Langmuir.

[32] Rosenthal I. (1978). Photochemical stability of rhodamine 6G in solution. Opt. Commun..

[33] Nie S., Emory S.R. (1997). Probing single molecules and single nanoparticles by surface-enhanced raman scattering. Science.

[34] Sliman O., Bumm L.A., Callaghan R., Blatchford C.O., Kerker M. (1983). Surface-enhanced Raman scattering by citrate on colloidal silver. J. Phys. Chem..

