# Supplementary material for: Silver Eco-Solvent Ink for Reactive Printing of Polychromatic SERS and SPR Substrates
Source: Sensors (Basel). 2018 Feb 9;18(2):521. doi: 10.3390/s18020521 (PMC5855186; doi:10.3390/s18020521)
Supplement: Supplementary file 1 [file sensors-18-00521-s001.pdf]

Article

Silver Eco-Solvent Ink for Reactive Printing of Polychromatic SERS and SPR Substrates

Mavlavi Dustov [1], Diana I. Golovina [1], Alexander Yu. Polyakov [1], Anastasia E. Goldt [1,2], Andrei A. Eliseev [1], Efim A. Kolesnikov [1], Irina V. Sukhorukova [3], Dmitry V. Shtansky [3], Wolfgang Grünert [4] and Anastasia V. Grigorieva [1,*]

[1] Department of Materials Science and Department of Chemistry, Lomonosov Moscow State University, Leninskie gory 1, bld. 73, 119991 Moscow, Russia; mr.mavlavi@mail.ru (M.D.); gol_dia@mail.ru (D.I.G.); a.yu.polyakov@gmail.com (A.Y.P.); chekanova@gmail.com (A.E.G.); eliseev@inorg.chem.msu.ru (A.A.E.); efimkolesnikov12@gmail.com (E.A.K.)

[2] Skolkovo Institute of Science and Technology, Skolkovo Innovation Center, bld. 3, 143026 Moscow, Russia

[3] National University of Science and Technology MISiS, Leninsky prospect 4, 119049 Moscow, Russia; irina_btnn@mail.ru

[4] Department of Chemistry and Biochemistry, Ruhr-Universität Bochum, Universitätsstraße 150, Bochum 44801, Germany; w.gruenert@techem.rub.de

* Correspondence: anastasia@inorg.chem.msu.ru; Tel.: +7-495-939-4609

Suggested concept of polycolor SERS platform production

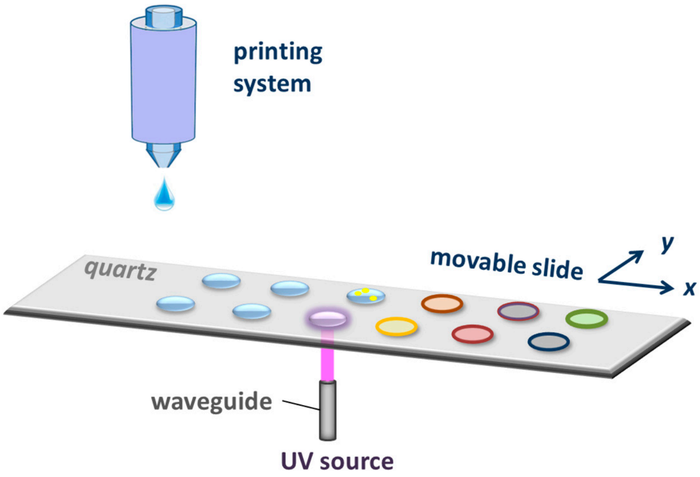

Figure S1. The hypothetic scheme for polycolor SERS platform production using photo-reactive silver complex inks.

Ink quality criteria

Stabilities of the model inks were estimated using standard physical and chemical methods such as time-resolved viscometry and Wilhelmi plate surface tension and transmittance analyses. The related key parameters of the liquid silver containing

compositions are given in Table S1. The experimental surface tension σ values for silver complexes in both aqueous and water-polyol media demonstrated a negative deviation of values from surface tension σ for pure dispersion media as elucidated theoretically [M. Rusdi, Y. Moroi, H. Nakahara, O. Shibata. Evaporation from water-ethylene glycol liquid mixture. Langmuir , 2005, **21**, 7308-7310].

The kinematic viscosity data collected for the various ink compositions of silver citrate and silver ammonia acetate complexes demonstrated an increase with EG percentage in both mixtures from nearly 6.5 to 12.0 cSt for the equivalent H_2O : EG mixture (Table S1). In contrast, the mixtures with larger volume percentage of EG exceeded the maximal viscosity parameter available for printing.

Measurement of kinematic viscosity was made with an Oswald-type glass capillary viscometer at 25°C. The capillary diameter was 131 mm, the kinematic viscosity was calculated according to $\upsilon = \frac{g \cdot K \cdot t}{9.807}$, where ν is the kinematic viscosity (mm^2/s), *K* is a viscometer constant of 0.277 mm^2/s^2, *t* – the time of liquid outflow time in seconds, *g* – the gravitational acceleration in m/s^2, and 9.807 m/s^2 is an instrument calibration constant which is equal to *g* constant in standard conditions. Parameter error value was estimated from 5 measurements.

Table S1 shows surface tension and kinematic viscometry experimental data for 0.1 M silver citrate complex in water – ethylene glycol media at different ratios of compounds.

Table S1. Characteristics of silver citrate complex in water-polyol (H_2O – EG) medias of different volumetric ratios.

Volumetric ratio	H_2O : EG	0.1 M $AgCit_n$ in H_2O : EG
	Surface tension σ, mN/m	
1 H_2O : 4 EG	51.5 ± 0.5	52.3± 0.7
1 H_2O : 3 EG	52.3± 0.6	53.5± 0.8
1 H_2O : 2 EG	53.8± 0.4	55.8± 0.6
1 H_2O : 1 EG	56.0± 0.7	56.8 ± 0.5
2 H_2O : 1 EG	59.6 ± 0.4	62.1± 0.6
3 H_2O : 1 EG	63.8± 0.5	64.7± 0.7
4 H_2O : 1 EG	64.4± 0.3	65.6 ± 0.4
	Kinematic viscosity υ , cSt	
1 H_2O : 1 EG	4.48 ± 0.02	4.25 ± 0.01
2 H_2O : 1 EG	2.22 ± 0.01	2.12 ± 0.01
3 H_2O : 1 EG	1.84 ± 0.02	1.87 ± 0.01
4 H_2O : 1 EG	1.69 ± 0.01	1.67 ± 0.02
